# Ways to study changes in psychosocial work factors

**DOI:** 10.5271/sjweh.4081

**Published:** 2023-02-27

**Authors:** Cécile RL Boot, Roosmarijn MC Schelvis, Suzan JW Robroek

**Affiliations:** 1 Amsterdam Public Health research institute, Societal Participation and Health, Amsterdam, The Netherlands; 2 Amsterdam UMC, VU University, Department of Public and Occupational Health, Amsterdam, The Netherlands; 3 Radboud University, Behavioural Science Institute, Nijmegen, The Netherlands; 4 Amsterdam UMC, University of Amsterdam, Department of Public and Occupational Health, Coronel Institute of Occupational Health, Amsterdam, The Netherlands; 5 Erasmus University Medical Center, Department of Public Health, Rotterdam, The Netherlands

Unfavorable psychosocial work factors are associated with poorer worker health, such as depression and cardiovascular disease (1). Most evidence is based on studies linking the exposure level at baseline to worker outcomes at follow-up (1). A next step to better understand the impact of psychosocial work factors on worker health is to investigate changes in exposure levels (2).

There is no consensus on how to operationalize changes in psychosocial work factors. This is not surprising for three reasons. Firstly, psychosocial work factors are most often measured by self-report (eg, 3, 4). This makes interpretation and generalization more difficult compared to other, more objectively measured exposure–response relationships such as, eg, exposure to chemicals. Due to these self-reports, measured differences in psychosocial factors may be biased and thus not always reflect a change in the work environment. Several attempts have been made to counteract this challenge in the field by using indicators from registers (5), cluster unit analyses (6), and job exposure matrices (7). Secondly, there is no consensus on which psychosocial work factors in what combinations are unfavorable for workers. For example, the Danish Psychosocial Work Environment Questionnaire (DPQ) covers as much as 38 psychosocial constructs (4). Knowledge is still limited: which ones are most important for worker health and which ones are of less importance. Because of this knowledge gap, we still do not know what the exposure–response relationship looks like. What is clear, though, is that exposure to high job strain (ie, high job demands in combination with low job control) is detrimental for worker health (1, 8). Thirdly, there is no consensus on cut-off scores for unhealthy psychosocial work factors, which makes it difficult to interpret changes in these factors and, thus, to compare results across studies.

To examine to what extent and in what manner changes in psychosocial work factors are being studied, we conducted a search in Pubmed on 25 January 2023 using the search terms ‘change’, ‘employees or workers’ and ‘work factors’ in the title or abstract, which yielded 7461 hits (table 1). The *Scandinavian Journal of Work, Environment and Health* (SJWEH) published 82 papers including these search terms. Of these 82 published papers, 11 studies analyzed changes in psychosocial work factors (9–19). In these SJWEH publications, we found two different approaches to investigate change in psychosocial work factors. Most studies focused on membership of a high risk group (9–14). First, they defined cut-offs based on the distribution of data points, and subsequently divided respondents into four groups based on their scores over time: stable unfavorable, worsening, improving, and stable favorable psychosocial work factors (9–12, 14), in which the ‘stable unfavorable’ and ‘worsening’ groups can be considered as high-risk groups. Moreover, many studies combined job demands and resources into one measure for job strain and analyzed changes in this composite score (11, 12), which was subsequently related to worker outcomes.

**Table 1:** Pubmed search: 25 January 2023

Total	*Published in Scandinavian Journal of Work, Environment and Health*
"change*"[title/abstract] AND ("employees"[title/abstract] OR "workers"[title/abstract]) AND ("psychosocial"[title/abstract] OR "strain"[title/abstract] OR "control"[title/abstract] OR "autonomy"[title/abstract] OR "demands"[title/abstract] OR "rewards"[title/abstract] OR "working conditions"[title/abstract] OR "work factors"[title/abstract])	"Scandinavian journal of work, environment health"[Journal] AND "change*"[title/abstract] AND ("employees"[title/abstract] OR "workers"[title/abstract]) AND ("psychosocial"[title/abstract] OR "strain"[title/abstract] OR "control"[title/abstract] OR "autonomy"[title/abstract] OR "demands"[title/abstract] OR "rewards"[title/abstract] OR "working conditions"[title/abstract] OR "work factors"[title/abstract])
7461 results	82 results
*Filter:* last 10 years: 3698 results	

As no golden route exists to analyze change in psychosocial work factors, in the following, we present some options, and describe pros and cons of these options. This editorial is a call not only to carefully think through how to define change in a study but also to share arguments for the choice of definition in the methods section and to be transparent about the underlying assumptions. The key issue to consider is the approach to study change in exposure. There are two different ways to study change. Firstly, studying changes in exposure levels (eg, an increase of 1 point) that will lead to an increase of the risk at individual level. Secondly, studying transitions in membership of high-risk groups. Below we will explain both options that we illustrate with examples.

**Option 1: Changes in exposure levels**

When a linear exposure–response relationship is assumed, every increase in exposure is associated with an increase in risk (15–17, 19). For example, Milner and colleagues analyzed how the psychosocial quality of a job was associated with mental health outcomes using longitudinal fixed effects regression models (16). The disadvantage of this method is that every change is considered equally relevant. This means that every unit of change (eg, 1 point or 1 standard deviation) in score has a similar effect on the outcome, independent of the baseline value. However, since psychosocial factors are often measured as ordinal variables, we know that the differences between the answering categories are not equal. For example, answering options in dimensions of the Copenhagen Psychosocial Questionnaire (3) are on a scale from 1 ‘always’ to 5 ‘never/hardly ever’, and a change in exposure from 1 ‘always’ to 2 ‘often’ versus from 2 ‘often’ to 3 ‘sometimes’ may have a different effect on the outcome (3). Most studies on changes focused on within-person changes, in our search we found only one study applying a population approach to study trends in psychosocial work factors over time (17).

**Option 2: Changes in high-risk group membership**

The second approach involves membership of a high-risk group by crossing a threshold or cut-off score. A worker can enter a high-risk group when crossing the threshold, even with a minor change in score. Yet, another person can remain in the low risk group with a larger change in score. When the threshold for high risk is for example 3.5, a worker with an increase of 2 points from 1.0 to 3.0 will not become at risk, whereas a worker with a score increase of 0.5 points from 3.1 to 3.6 will become at risk. The increase itself, 2 points versus 0.5 points is not informative here; what matters is whether the threshold from the low risk to the risk group is crossed. In this case, it is important to consider that not all workers crossing the threshold will have a relevant change in score, as the group of workers very close to the threshold will only need a little change to cross it.

When taking the high-risk approach, it is important to define what we consider a high risk. Ideally, the relevant cut-off scores are known. Alternatively, the high-risk group can be defined based on the distribution of scores within the population, based on eg, the median (9–11, 14, 18), (upper) tertile (13) or any exposure (versus no exposure) (10). This method using the median as a cut-off is most often used in studies on change published in SJWEH. For example, Saastimoinen and colleagues performed a median split for job demands and control variables separately, which enabled them to construct a variable for job strain based on high/low demands/control (9). Too and colleagues analyzed data from four waves of the Whitehall study based on a tertile split and showed how job control could vary between low, medium, and high levels over time (13). With the cut-off, one takes into account the distribution of scores within a population, and the researchers define which percentage of the population is at high risk. However, depending on the population, the cut-off score for being at high risk may differ largely between populations. This makes it difficult to compare exposure–response associations between studies. When demand and control variables are combined into a job strain measure (eg, 9), the comparison becomes even more challenging. Moreover, how sure can we be that a specific percentage of our research population is at risk? By assuming that by definition 50% (median split), 33% (tertile split), or 25% (quartile split) of the sample is at risk, the exposure prevalence is defined by the researcher and a similar exposure prevalence across studies may hide completely different exposure patterns.

Both the changes in exposure levels and the changes in high-risk group membership approaches have their pros and cons (table 2). In both approaches, the comparability across studies is an issue as (i) different questionnaires and rating scales are used to measure psychosocial work factors and (ii) different choices are made for defining levels of psychosocial work factors as unfavorable (eg, different cut-off values).

**Table 2:** Overview of pros and cons of the change in exposure levels approach and the high-risk group approach

	Advantage	Disadvantage
Changes in exposure levels	More detailed information about time trends	Every change considered equally relevant for outcome
Changes in high-risk group membership	Takes into account ordinal scales of current questionnaires	Switch between high and low risk groups may be due to random measurement errors for scores close to the threshold

Analyzing changes in psychosocial work factors requires clarity on the approach. When taking the changes in exposure levels approach, an interpretation of the meaning of a certain change (eg, from 1 to 2) is needed. When taking the high-risk approach, transparent choices have to be made to define the high risk group(s). This is important because these choices can heavily influence the conclusions. Hence, we strongly recommend to explain the choices and the underlying assumptions made to enhance the interpretation of the results. In that way, the evidence becomes more comparable which will improve our understanding of exposure–response associations in studies on psychosocial factors at work and worker health.
